# Evaluation of input data modality choices on functional gene embeddings

**DOI:** 10.1093/nargab/lqad095

**Published:** 2023-11-02

**Authors:** Felix Brechtmann, Thibault Bechtler, Shubhankar Londhe, Christian Mertes, Julien Gagneur

**Affiliations:** TUM School of Computation, Information and Technology, Technical University of Munich, Garching, Germany; Munich Center for Machine Learning, Munich, Germany; TUM School of Computation, Information and Technology, Technical University of Munich, Garching, Germany; TUM School of Computation, Information and Technology, Technical University of Munich, Garching, Germany; TUM School of Computation, Information and Technology, Technical University of Munich, Garching, Germany; Munich Data Science Institute, Technical University of Munich, Garching, Germany; Institute of Human Genetics, School of Medicine, Technical University of Munich, Munich, Germany; TUM School of Computation, Information and Technology, Technical University of Munich, Garching, Germany; Institute of Human Genetics, School of Medicine, Technical University of Munich, Munich, Germany; Computational Health Center, Helmholtz Center Munich, Neuherberg, Germany

## Abstract

Functional gene embeddings, numerical vectors capturing gene function, provide a promising way to integrate functional gene information into machine learning models. These embeddings are learnt by applying self-supervised machine-learning algorithms on various data types including quantitative omics measurements, protein–protein interaction networks and literature. However, downstream evaluations comparing alternative data modalities used to construct functional gene embeddings have been lacking. Here we benchmarked functional gene embeddings obtained from various data modalities for predicting disease-gene lists, cancer drivers, phenotype–gene associations and scores from genome-wide association studies. Off-the-shelf predictors trained on precomputed embeddings matched or outperformed dedicated state-of-the-art predictors, demonstrating their high utility. Embeddings based on literature and protein–protein interactions inferred from low-throughput experiments outperformed embeddings derived from genome-wide experimental data (transcriptomics, deletion screens and protein sequence) when predicting curated gene lists. In contrast, they did not perform better when predicting genome-wide association signals and were biased towards highly-studied genes. These results indicate that embeddings derived from literature and low-throughput experiments appear favourable in many existing benchmarks because they are biased towards well-studied genes and should therefore be considered with caution. Altogether, our study and precomputed embeddings will facilitate the development of machine-learning models in genetics and related fields.

## Introduction

Annotations of gene functions, i.e. the biological processes gene products are involved in (1), are important to help understand how genetic variations affect molecular and cellular physiology. Therefore, functional annotations of genes are instrumental for delineating and computationally modelling genotype-to-phenotype relationships. However, functional annotations of genes are typically provided in the form of complex data structures, such as free text and ontology-based annotations, that cannot be easily used in machine learning models.

Representation learning techniques, which allow mapping heterogeneous and complex data structures including graphs and words (2,3) to numeric vectors called embeddings, can address this issue. Studies have demonstrated that functional gene embeddings can be derived from a variety of data types reflecting gene function, including gene expression (4–6), CRISPR screens (7), protein sequence (8), protein–protein interaction networks (9–11), but also occurrences in scientific literature (12) and Gene Ontology (13) annotations (14). These studies have shown that the obtained embeddings have predictive power for a wide range of downstream prediction tasks including the prediction of disease-gene lists (9,12), Gene Ontology annotations (6,7), gene-phenotype associations (15), but also gene-gene interactions (5,11,14) and subcellular localizations of gene products (10). These results are appealing because they suggest that precomputed functional gene embeddings could become effective resources for integrating gene function information into machine learning models. So far much attention has been put to comparing the different embedding algorithms and their flexibility to incorporate various data modality types (9,11). However, comparisons of the impact of choosing alternative input data modalities when building functional gene embeddings with respect to downstream applications are lacking.

In particular, the fact that some input data modalities as well as the downstream evaluation tasks are biassed for the most studied genes (16) could confound the evaluation of functional gene embeddings. This is concerning because functional gene embeddings could specifically underperform on the least studied genes. Hence, functional gene embeddings are prone to suffer from and further contribute to the pervasive street-light effect, i.e. biassing research attention to the already most studied genes (17,18). With 95% of all life science publications devoted to about 5000 human proteins in 2018 (19), annotation inequality is strong and could substantially affect gene embeddings based on literature and manually curated annotations (20,21). Annotation inequality also affects embeddings derived from experimentally reported protein–protein interactions (PPI), as those often stem from low-throughput experiments focussing on genes of interest (20,21). Additionally, the reliability of published interactions reduces the more studied a protein is (22). This is further concerning as functional gene embeddings created from PPI data could propagate this reduced reliability further to downstream applications. Evaluation tasks that are most likely to be biassed by annotation inequality include predictions of curated disease-gene lists and Gene Ontology annotations.

Here we evaluate the utility of alternative input modalities for precomputed functional gene embeddings as well as the issue of annotation inequality bias. To do so, we generate embeddings that are either based on systematic genome-wide assays and therefore devoid of annotation inequality bias, or derived from text mining, manually curated datasets, or low-throughput experiments. Next, we evaluate the utility of using these precomputed embeddings as gene features to predict gene annotation and disease genes with off-the-shelf machine-learning algorithms. We compare them against dedicated state-of-the-art prediction models when available. Finally, we propose two new predictive tasks based on genome-wide association studies which aim to circumvent annotation inequality bias in the evaluation.

## Materials and methods

### Gene universe and gene identifier mapping

We considered all 19385 human protein-coding genes from STRING version 11.5 (23) and used the Ensembl gene IDs (24) as unique identifiers. Unless stated otherwise, gene or protein identifiers of external sources were mapped to Ensembl gene IDs using BioMart (24) and gene names were mapped to Ensembl gene IDs using a map obtained from the HUGO Gene Nomenclature Committee (HGNC, https://www.genenames.org/, downloaded on 13 June 2022).

### Omics embedding

To create the Omics embedding, we integrated gene expression across human tissues from the GTEx project (25) and two recently published datasets: a genome-wide essentiality screen from DepMap (26) and a functional gene embedding derived from a language model trained on hundreds of millions of protein sequences (8). For the GTEx gene expression dataset, we downloaded the transcript per million (TPM) matrix and subset it to only contain protein-coding genes, resulting in a matrix of 18 523 genes and 17 382 samples. Additionally, the TPM values were log-transformed and mean-centred per gene.

Essentiality estimates computed using CERES (27) were downloaded from the DepMap Project Achilles 18Q3 release (26) for 17585 genes across 485 different cancer cell lines were downloaded from the DepMap portal. The same preprocessing as in the study by Wainberg *et al.* (28) was applied, that is, we removed global technical variations by removing the first four principal components computed on the 411 olfactory genes from the data. To avoid potential multicollinearity, the values of four cell lines were removed. A list of olfactory receptor genes is available from the original publication (28). Protein sequences were queried from the Ensembl database using BioMart (24). The query comprised all protein-coding genes as selectable in the ‘filters’ section. The query result was then matched using the list of protein-coding genes described above resulting in 18320 protein sequences. For each protein sequence, a protein embedding was computed using the ProtT5 model (8). To achieve this, first, every sequence was embedded using the ProtT5 model yielding an embedding per residue. Then, these embeddings were averaged across all residues to obtain one embedding vector per protein as previously described (8). This resulted in a protein embedding with a dimensionality of 1024 by 18 320 proteins.

The aforementioned omics data sets were modelled with a Variational Deep Tensor Factorization Model, an extension of a previously published method. Deep Tensor Factorisation models approximate data matrices by learning embeddings and an aggregation function. Assume matrix **X** with values ${x}_{ij}$ for gene *i* and sample *j*. A tensor factorisation model learns an embedding **a***_i_*for every gene *i* and **b***_j_* for every sample *j* and a function *f* combining the two embeddings to predict a reconstruction of ${x}_{ij}$ denoted ${\hat{x}}_{ij}$:


\begin{equation*}{\hat{x}}_{ij}\ = f({{{\bf a}}}_i,{{{\bf b}}}_j).\end{equation*}

The function *f* is parameterized as a feed-forward neural network on the concatenation of the inputs **a***_i_*,**b***_j_* with 3 layers and a leaky-ReLU activation function between the layers. The architecture was not subject to optimisation. All model parameters, that are, the embeddings **A** and **B**, and the parameters of the function *f* are updated by gradient descent, minimising the mean squared error loss function between the observation ${x}_{ij}$ and the reconstruction ${\hat{x}}_{ij}$. This model can deal with missing values simply by not summing over them in the loss function. The dataset can be split into training and validation sets by randomly splitting the index pairs (*i*,*j*) into these sets.

To learn a variational embedding from multiple datasets, we made three modifications to this model.

First, we generalised the model to multiple datasets by sharing the gene embedding **A** between models yet learning independent sample embeddings and independent functions for each dataset. Assume three datasets **X^(1)^**, **X^(2)^** and **X^(3)^** are given, we wrote the models as:


\begin{equation*}\begin{array}{@{}*{1}{l}@{}} {{{\widehat {{x}^{\left( 1 \right)}}}}_{ij} = {f}_1({{{\bf a}}}_i,{{{\bf b}}}_j^{({{\bf 1}})})}\\ {{{\widehat {{x}^{\left( 2 \right)}}}}_{ij^{\prime}} = {f}_2({{{\bf a}}}_i,{{{\bf b}}}_{j^{\prime}}^{({{\bf 2}})})}\\ {{{\widehat {{x}^{\left( 3 \right)}}}}_{ij^{\prime\prime}} = {f}_3({{{\bf a}}}_i,{{{\bf b}}}_{j^{\prime\prime}}^{({{\bf 3}})})} \end{array}\end{equation*}

where the embedding of the *i*th gene **a***_i_* is shared across all datasets, *f_1_*, *f_2_* and *f_3_* denote the respective dataset-specific functions and **b***_j_***^(1)^**, **b***_j’_***^(2)^** and **b***_j’’_***^(3)^***_’_* denote the embeddings of samples *j*, *j’* and *j’’* respectively (Supplementary Figure S1).

Second, we observed while developing our model, using UMAP’s that our embeddings learnt gene-inclusion biases of the different datasets. To mitigate this effect, we used the gradient reversal layer, a technique from domain adaptation learning, introduced by Ganin and Lempitsky (29). This layer prevents the embedding from learning in which dataset a gene was present. A small two-layer neural network, called domain classifier, is trained to predict which gene is missing. During backpropagation, the gradient between the network, predicting which value is missing, and the embedding is reversed, so that the embedding is optimised under the opposite objective, namely that we cannot predict from the embedding which gene is missing in which dataset.

Third, to cope with fitting instabilities that we encountered during early investigations, we considered a variational version of the model.

Specifically, we considered a data generative model in which dataset-specific gene embeddings $a_{ik}^{( l )}$ are randomly drawn given a common gene embedding $a_{ik}$ according to:


\begin{equation*}a_{ik}^{( l )} \sim \mathcal{N}\left( {a_{ik},\sigma_{ik}^2 + {v}_{ik}^{\left( l \right)}} \right)\end{equation*}

where $\mathcal{N}$ denotes the Gaussian distribution, $a_{ik}$ is the functional embedding value for gene *i* and embedding dimension *k*, ${\sigma }^2_{ik}$ is a minimal common variance term for all datasets, and ${v}_{ik}^{( l )}$ is a further dataset-specific variance term. The reconstructed dataset was then obtained according to:


\begin{equation*}{\widehat {{x}^{\left( l \right)}}}_{ij} = {f}_l\left( {{{{\bf a}}}_i^{(l)},{{{\bf b}}}_j^{(l)}} \right),\end{equation*}

where **a***_i_*^(l)^ is the vector over all embedding dimensions *k* of $a_{ik}^{( l )}$, and **b***_j_*^(l)^ is the embedding of the *j*th sample of dataset $l$.

To fit the model parameters we maximised the evidence lower bound (ELBO) of the model given by minimising:


\begin{eqnarray*} && \mathop \sum \limits_l \mathcal{L}\ ({{\boldsymbol{X}}}^{\left( l \right)}\ |\ \widehat {{{\boldsymbol{X}}}^{\left( l \right)}})\ + \ {D}_{KL}(\mathcal{N}\left( {{a}_{ik},\ \sigma _{ik}^2} \right)\ ||\ \mathcal{N}\left( {0,\ 1} \right))\nonumber\\ && \quad +\, \mathop \sum \limits_l {D}_{KL}\left(\mathcal{N}\left( {0,\ v_{ik}^{\left( l \right)}} \right)\ ||\ \mathcal{N}\left( {0,\ v_{prior}^{\left( l \right)}} \right)\right),\ \end{eqnarray*}

where $\mathcal{L}( {{X}^{( l )}{\mathrm{|}}\widehat {{{\boldsymbol{X}}}^{( l )}}} )$ is the reconstruction loss for dataset $l$ (negative log-likelihood) and *D_KL_*(*p* || *q*) denotes the Kullback–Leibler divergence between two distributions *p* and *q*. The parameters of the models are the common gene embedding **A**, the common variance terms **Σ**, the dataset-specific variance terms **V**^*(l)*^, the sample embeddings **B**^(^*^l^*^)^ and the parameters of the reconstruction functions *f_l_*, for all values of $l$. The parameters were fitted by maximising the evidence lower bound using blockwise gradient descent, where the blocks were constructed per dataset. Each block included the dataset-specific sample embeddings **B**^(^*^l^*^)^ and the reconstruction function *f_l_*. Furthermore, each block contained the common gene embedding **A**. The domain classifier model was also contained within every block. Its loss function was added to the overall loss function. All model parameters were initialised randomly except for the dataset-specific sample embeddings which were initialised using principal components of the respective datasets. Then, the model parameters were updated using Adam (30). For stochastic gradient descent, larger datasets need to be divided into more batches than smaller datasets. This creates imbalanced influences of the datasets on the gene embedding updates. For example, the GTEx dataset contained approximately 17 000 samples and was split into 294 batches, whereas the DepMap dataset contained 485 cell lines and was split into only 8 batches. This large discrepancy in the number of batches resulted in the GTEx dataset dominating the gene embedding updates. To overcome this, we scaled the learning rate of the embedding layer for each dataset relative to the number of batches. By monitoring the validation error, we found that dividing the global learning rate by the square root of the number of batches per dataset resulted in the fastest convergence and the lowest validation error compared to other scaling schemes evaluated.

The hyperparameters of the models were the three dataset-specific prior variances $v_{prior}^{( l )}$ for each dataset $l$ (i.e. GTEx, DepMap and ProtT5). De facto the dataset-specific prior variances allow adjusting the relative influence of each dataset on fitting the common gene embedding **A**. Hyperparameter optimization was performed using Optuna (31). We found that using reconstruction loss as an evaluation metric for hyperparameter optimisation gave poor results in downstream tasks. We also investigated a multi-objective hyperparameter optimization (32) but it was not robust. Instead, we opted to maximise the area under the Precisions-Recall Curve (auPRC) of recalling high-confidence STRING edges (STRING score > 0.9, see below) when ranking gene pairs by the Euclidean distance between their embedding vectors. Using STRING to learn hyperparameters may in principle bias our results. However, it is reasonable to think that no gene-level information specific to STRING was leaked into the Omics embedding this way because hyperparameter optimization only concerned three scalars, namely the three dataset-specific prior variances. We further set the dataset-specific sample embedding dimensions to 256 for GTEx, 128 for protT5 and 64 for depMap after initial investigations using PCA and cross-validation. The dataset-specific sample embedding dimensions were not further optimised.

### STRING embeddings

As a source of functional protein associations and interactions, the STRING database version 11.5 was used. This database infers functional associations from different data sources including gene co-expression, high-throughput laboratory experiments, genetic evidence, curated PPIs, pathway databases and automated literature text mining. We downloaded the human part from the STRING webpage and mapped the STRING identifiers to Ensembl gene IDs using the mapping table provided by STRING.

Based on the STRING dataset, we created two networks that we refer to as STRING and STRING experimental. STRING comprised the full human network of STRING with 5 969 249 edges and 19 385 genes. For STRING experimental, we removed evidence derived from manually curated databases and text mining, resulting in a network with 1620401 edges and 18711 nodes. The filtering was performed by removing all evidence channels that contained database annotations or literature both transferred from other species and directly annotated to human PPIs. To obtain a single combined score for the new network from the remaining channels we followed the description from the STRING documentation (https://string-db.org, see the FAQ entry titled ‘How are the scores computed?’, last accessed 7^th^ September 2023). In brief, we removed for each individual channel the prior (*p*= 0.041) stated in the documentation and combined all channels using the equation $S = 1 - \mathop \prod \limits_c ( {1 - {S}_c} )$ where c is the channels and *S_c_* the scores of each channel. Subsequently, we injected the prior back into the combined score as stated in the STRING documentation. Consequently, edges purely based on text mining or databases were removed entirely.

We generated embeddings for each network by applying the graph embedding algorithms node2vec (2) and VERSE (33) to the networks. We ran both algorithms with the following parameters: 128 embedding dimensions for both algorithms and for node2vec a random walk length of 80, a number of walks per source of 10, one SGD epoch, the return hyperparameter set to 0.3 and the inout hyperparameter set to 1. We ran VERSE with the same number of dimensions of 128 and the damping factor α of the internal Personalised PageRank set to 0.85. Furthermore, three negative samples were drawn per positive sample. We later concatenated the node2vec and VERSE embeddings to obtain one embedding for STRING and one for STRING experimental.

### Polygenic Priority Score (PoPS) embeddings

The Polygenic Priority Score (PoPS) features were downloaded from https://www.finucanelab.org/data on 5 August 2022 (34). The gene features of the PoPS study are based on multiple gene expression experiments and partially manually curated datasets, including pathway annotations and PPI databases. To create a version of the PoPS feature matrix containing data only from experiments, and excluding data from manually curated sources we limited the PoPS feature matrix to contain only experimental (expression) features.

### Gene-list predictions

We obtained curated gene lists for six diseases (Table 1, first rows). We further considered the set of positive and negative cancer genes provided by the EMOGI study (35). Additionally, trait-gene associations were obtained from the Genebass database, which contains exome-based single-variant and gene-based association studies for 394841 individuals. We extracted the gene-based results using a burden test and SKAT-O and filtered for significant associations using the suggested *P*-value cut-off of 6.7 × 10^−7^ and 2.5 × 10^−7^ for the burden test and SKAT-O respectively (36). As recommended by Karczewski *et al.* (36), we further filtered to a high-quality set of genes with at least 20-fold coverage and at least one phenotype with an expected allele count ≥50 for pLoF (7296 genes) and missense (15 943) using the QC data provided at gs://ukbb-exome-public/500k/qc/gene_qc_metrics_ukb_exomes_500k.mt. Further filtering was performed to include only traits associated with at least 30 genes, resulting in a set of 61 traits. The similarity between these traits was quantified using Jaccard indices. Next, one representative trait was chosen from clusters of highly similar traits (Jaccard index > 0.3), resulting in the final set of 34 unrelated traits (SupplementaryFigure S2). All used traits are listed in Supplementary Table 1.

**Table 1.** Gene lists used for classification benchmarks

Trait	Genes	Source
Mitochondrial diseases (MITO)	400	Schlieben *et al.*, 2020 (37)
Ophthalmology (OPH)	365	Frésard *et al.*, 2019 (38)
Inborn Errors of Immunity (IEI)	364	Rapaport *et al.*, 2021 (39)
Neurology (NEU)	284	Frésard *et al.*, 2019 (38)
Neuromuscular (NM)	131	Gonorazky *et al.*, 2019 (40)
Epilepsy (EP)	83	Wang *et al.*, 2017 (41)
Cancer driver gene (CG)	783	Schulte-Sasse *et al.*, 2021 (35)
Genebass 34 traits	30–165	Karczewski *et al.*, 2021 (36)

For each gene list, including the cancer gene list, eXtreme Gradient Boosting (XGBoost) models were trained for each trait gene list in 5-fold cross-validation to predict whether a gene was contained in one of those lists. To do so, all genes annotated or significantly associated with a disease or trait were treated as the positive set and all other protein-coding genes as the negative set. Therefore, for each disease, the negative set may contain yet-to-be-discovered disease genes. We trained the XGBoost model in random five-fold cross-validation. On the PoPS and PoPS experimental feature matrices, logistic regression models with elastic regularisation were used in place of XGBoost, as fitting an XGBoost model was infeasible due to the large dimensionality of the input data.

Hyperparameters were optimised for the XGBoost model using nested cross-validation. Specifically, we tuned the L1 and L2 regularisation parameters in the XGBoost loss function over a log-uniform grid from 10^−2^ to 10^5^ for both parameters.

The models were evaluated by computing the precision-recall curves and the average precision on each validation fold. The folds were set to be the same across embedding models. Table 1 provides an overview of all gene lists that we considered.

### Human Phenotype Ontology (HPO) predictions

Gene-phenotype annotations were downloaded from the Human Phenotype Ontology (HPO) webpage (http://purl.obolibrary.org/obo/hp/hpoa/genes_to_phenotype.txt, downloaded on 13 June 2022) (42). NCBI gene identifiers were mapped to Ensembl gene IDs. We limited the HPO terms to those having at least 20 genes annotated, resulting in a final set of 1994 HPO terms.

The HPO gene-phenotype associations were multi-hot encoded, that is, the label matrix entry *y_ij_* was set to 1 if an association was reported for phenotype *i* and gene *j* and 0 otherwise. Genes were randomly split into a 70% training and a 30% test set. A two-layer neural network was trained to predict the HPO label matrix using the functional gene embedding as an input on the training genes. The model was fitted by minimising the binary cross-entropy using the Adam optimiser (30) for a fixed number of 500 epochs. Predictions from HPOFiller (43) were used as provided by the authors (https://doi.org/10.6084/m9.figshare.13487277, dataset posted on 24 December 2020).

### Genome-wide association study signal predictions

The Pan UKB study (44) obtained genotyping data from the UKBiobank (45) and ran genome-wide association studies (GWAS) for 7228 phenotypes. We selected 22 maximally independent traits (traits showing low correlations with one another) from all available blood biomarkers from the Pan-UKB phenotype manifest using the ‘in_max_independent_set’ indicator (44). The 22 selected traits are listed in Supplementary Table S2.

The GWAS summary statistics were processed through MAGMA (46) using default parameters and the reference set provided by the PoPS team (https://www.finucanelab.org/data downloaded on the 5 August 2022). For a given trait, MAGMA summarises gene–trait associations in one z-score per gene. We formulated the model for predicting the z-scores obtained by MAGMA in the following way:


\begin{equation*}{\boldsymbol{y}} = {{\bf C}}\alpha + {{\bf X}}\beta + \varepsilon ,\varepsilon \sim MVN\left( {0,{{\bf R}}} \right)\end{equation*}

where **X** is the functional gene embedding, **C** are covariates and the residuals $\epsilon$ follow a multivariate normal distribution *MVN*(0, **R**), with a covariance matrix **R** designed to account for the linkage disequilibrium between nearby genes computed from a reference panel. To fit an Elastic Net model, we projected the prediction target $y$ using a Cholesky decomposition of the covariance matrix **R**, to find a formulation in which the residuals *ϵ*’ follow a multivariate normal distribution with a diagonal covariance matrix.


\begin{equation*}{\boldsymbol{y^{\prime}}} = {{\bf C}}\alpha ^{\prime} + {{\bf X}}\beta ^{\prime} + \varepsilon ^{\prime}, \varepsilon ^{\prime} \sim MVN\left( {0,{{\bf I}}} \right).\end{equation*}

We trained the Elastic Net model to predict the projected z-score from the functional gene embeddings **X** and six additional covariates **C**. The covariates included gene density, effective gene size and inverse of the mean minor allele count (MAC) of SNPs in the gene as well as the log of these variables as computed by MAGMA. Leave-One-Chromosome-Out (LOCO) cross-validation was used on the 22 autosomes to evaluate the performance of the functional gene embedding on each trait. To achieve this, one chromosome was held out for the evaluation and the model was fitted on the remaining 21 chromosomes. Hyperparameters were determined using grouped folds of the 21 training chromosomes. After fitting the model and computing the predictions, the predictions were transformed back to the original MAGMA scale and compared with the targets. To compare the performance of the embeddings, we computed the coefficient of determination increase $\Delta {R}^2$ defined as:

$\Delta {R}^2 = {R}^2 - {\mathrm{\ }}R_0^2$ .

The coefficient of determination of the null model $R_0^2$ was computed by performing the training task on covariates only.

We report the wall timings in all cases. The times reported are for each individual trait. Elastic Net regression for all embeddings was run on a 16-core machine with 250 Gigabytes of RAM for the PoPS feature matrix and 30 Gigabytes of RAM for the other embeddings.

### PubMed counts

To obtain the number of publications per gene, we downloaded the tables gene2ensembl.gz and gene2pubmed.gz from PubMed (https://ftp.ncbi.nih.gov/gene/DATA/) on 28 September 2022.

## Results

### Generation of functional gene embeddings

**Figure 1.**
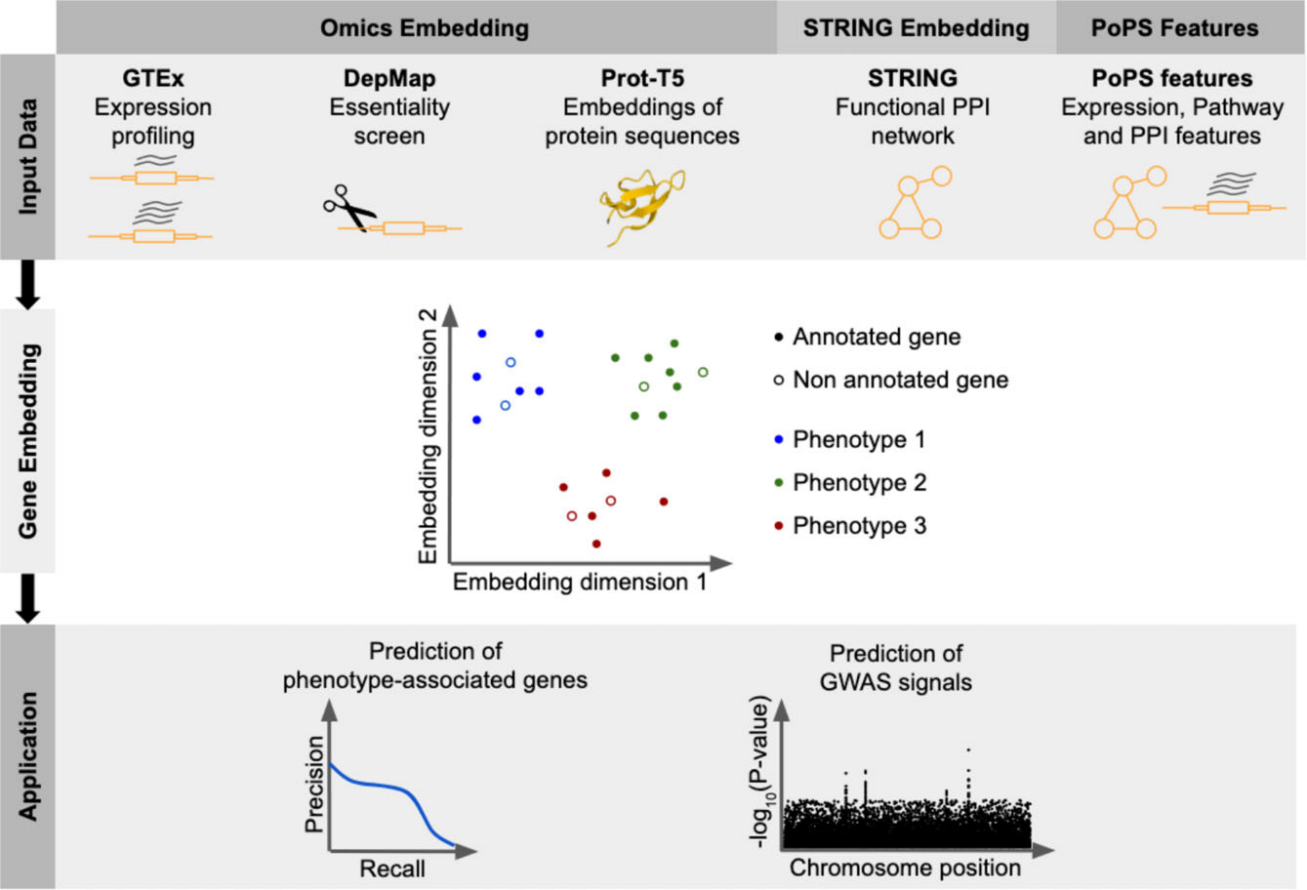
Study overview. Functional gene embeddings were created from multiple data sources. The Omics embedding was created from genome-wide assays: transcriptomics, CRISPR screens and protein sequence. The STRING embeddings were created by running graph embedding algorithms on two STRING networks (complete network and network based on experimental evidence only). We further included the features from the PoPS study, a study that aimed at predicting genome-wide association study signal from gene expression, pathway annotation and protein–protein interactions (34). All functional gene embeddings were benchmarked on various prediction tasks, including the prediction of phenotype-associated genes as well as predicting scores derived from large-scale genome-wide association studies.

We created multiple functional gene embeddings (Figure 1 and Table 2). The first embedding, which we call the Omics embedding, was derived from genome-wide high-throughput experimental data. This Omics embedding integrates gene expression across human tissues from the GTEx study (25) and two recently published datasets: a genome-wide essentiality screen from DepMap (26) and an embedding derived from a language model trained on hundreds of millions of protein sequences (8). One technical challenge we faced was that the sets of measured genes were not the same for all input datasets. To cope with dataset-specific missing values, we modified the deep tensor factorisation model by Trofimov *et al.* (6). We further generated two embeddings from the functional PPI network STRING (23) using graph embedding algorithms (Materials and Methods). The first STRING embedding used the entire STRING network, including evidence from the literature. The second STRING embedding, which we named STRING experimental, was based on PPIs derived from experimental evidence only. As we later compare our embeddings on GWAS signal prediction tasks introduced by Weeks et al., we obtained their gene features and named the corresponding embeddings after their method Polygenic Priority Score, or PoPS (34). The gene features of the PoPS study are based on multiple gene expression experiments, partially manually curated datasets including pathway annotations and protein–protein interaction databases. Similar to the STRING-based embeddings, we created two sets of PoPS features: (i) the full feature set and (ii) a subset containing only gene expression data, which we named PoPS experimental. For simplicity, we refer to the PoPS features and the PoPS experimental features also as functional gene embeddings. All of the embeddings were compared on multiple tasks, ranging from disease-gene list predictions to predictions of gene-phenotype links and predictions of GWAS signals.

**Table 2.** Functional gene embeddings and feature sets of different dimensions were created from multiple data sources

Embedding or gene features	Dimension	Supplementary table	Data source
Omics	256	Table S3, S4	GTEx, DepMap, ProtT5
STRING	256	Table S5	All edges of the STRING network
STRING experimental	256	Table S6	Edges of the STRING network derived from all sources of evidence except databases and literature mining
PoPS	57743		77 gene expression datasets, biological pathways curated for DEPICT from KEGG, GeneOntology, Reactome and the Mouse Genome databases, InWeb_IM protein–protein interaction (PPI) network
PoPS experimental	40547		77 gene expression datasets

### Disease–gene prediction

**Figure 2.**
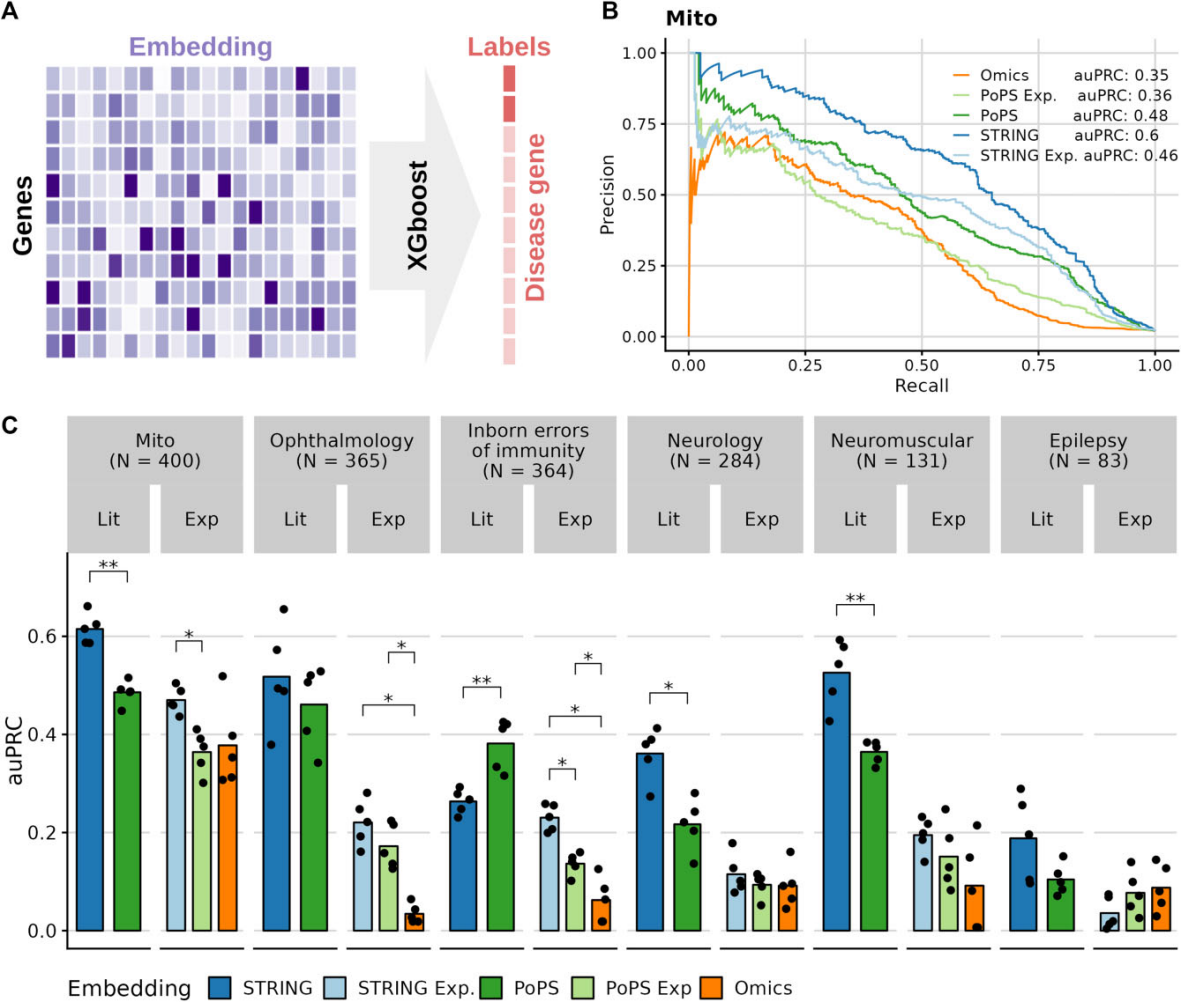
Disease–gene prediction. (**A**) We predicted disease–gene lists using XGBoost models trained on each embedding. Each gene associated with a disease was a true observation and all other protein-coding genes served as a negative set. (**B**) Precision plotted against recall for the mitochondrial disease–gene list. (**C**) Predictive performance (area under the precision-recall curve, auPRC) for each trait and embedding. Each dot corresponds to one cross-validation fold and the bar represents the mean. All statistically significant pairwise comparisons are marked with asterisks (two-sided Wilcoxon test, * *P*< 0.05, ** *P* < 0.01).

As a first benchmark task, we evaluated whether our embeddings were predictive for disease genes. To do so, we obtained six curated disease-gene lists and trained an XGBoost (47) model in five-fold cross-validation, predicting for each gene whether it was part of each list (Supplementary Tables S7–S12). For each disease, we treated all non-annotated genes as negative instances (Figure 2A). Hence, for each disease, the negative set probably contains disease genes that are yet to be discovered. We opted for XGBoost as it is a simple-to-apply algorithm implementing gradient-boosted trees. It is regarded as an off-the-shelf tool in contemporary machine learning because it performs well for a wide set of problems while not requiring user intervention or model architecture design (47). We used XGBoost to assess the performance of naïve yet realistic modelling attempts.

On the mitochondrial disease-gene list, the embedding of STRING achieved the highest area under the precision-recall curve (Figure 2B). Across all six disease-gene lists, the embeddings based on curated databases or literature mining (STRING and PoPS) generally outperformed the embeddings based on experimental data (STRING experimental, PoPS experimental and Omics; Figure 2C, Supplementary Tables S7–S12). We did not find a consistent difference in performance between the STRING and the PoPS embedding on this benchmark (Figure 2C). However, with only 256 dimensions for the STRING embedding in comparison to over 57 000 dimensions for PoPS, the STRING embedding appears advantageous on disease-gene list prediction according to this benchmark.

Among the embeddings based on experimental data only, there was also no clear winner. Only for the Ophthalmology and Inborn Errors of Immunity gene lists, the Omics embedding was significantly worse than the others. Further, STRING experimental and PoPS experimental only differed significantly for inborn errors of immunity. Again, the STRING experimental embedding, which has only 256 dimensions, performed advantageously on this benchmark in comparison to the PoPS experimental embedding with over 40 000 dimensions.

The predictions made using the Omics embeddings are particularly appealing because this embedding is based on neither literature nor single-gene experiments. Epilepsy was an interesting use case because it had the fewest annotated genes. Remarkably, among the five highest-ranked novel predictions, four were established after 2017 as epilepsy-associated genes (Table 3). The other gene, *SLC4A10*, was not part of the ground truth dataset, even though it was reported in 2008 by Gurnett and colleagues (48). These results illustrate the utility of the Omics embedding for discovering novel disease genes.

**Table 3.** Top five Epilepsy gene predictions based on the Omics embedding not annotated in the ground truth dataset from 2017 by Wang *et al.* (41)

Gene	Evidence for a role in epilepsy
*CNKSR2*	Higa *et al.*, 2021 (49)
*ATP2B2*	Rahimi *et al.*, 2022 (50)
*SLC4A10*	Gurnett *et al.*, 2008 (48)
*TUBB4A*	Sarkar *et al.*, 2022 (51)
*CACNG2*	Schirmer *et al.*, 2022 (52)

All five could be confirmed by further investigating literature. Of those, four publications were more recent than our ground truth dataset.

### Functional gene embeddings allow rapid development of predictive models with state-of-the-art performance

**Figure 3.**
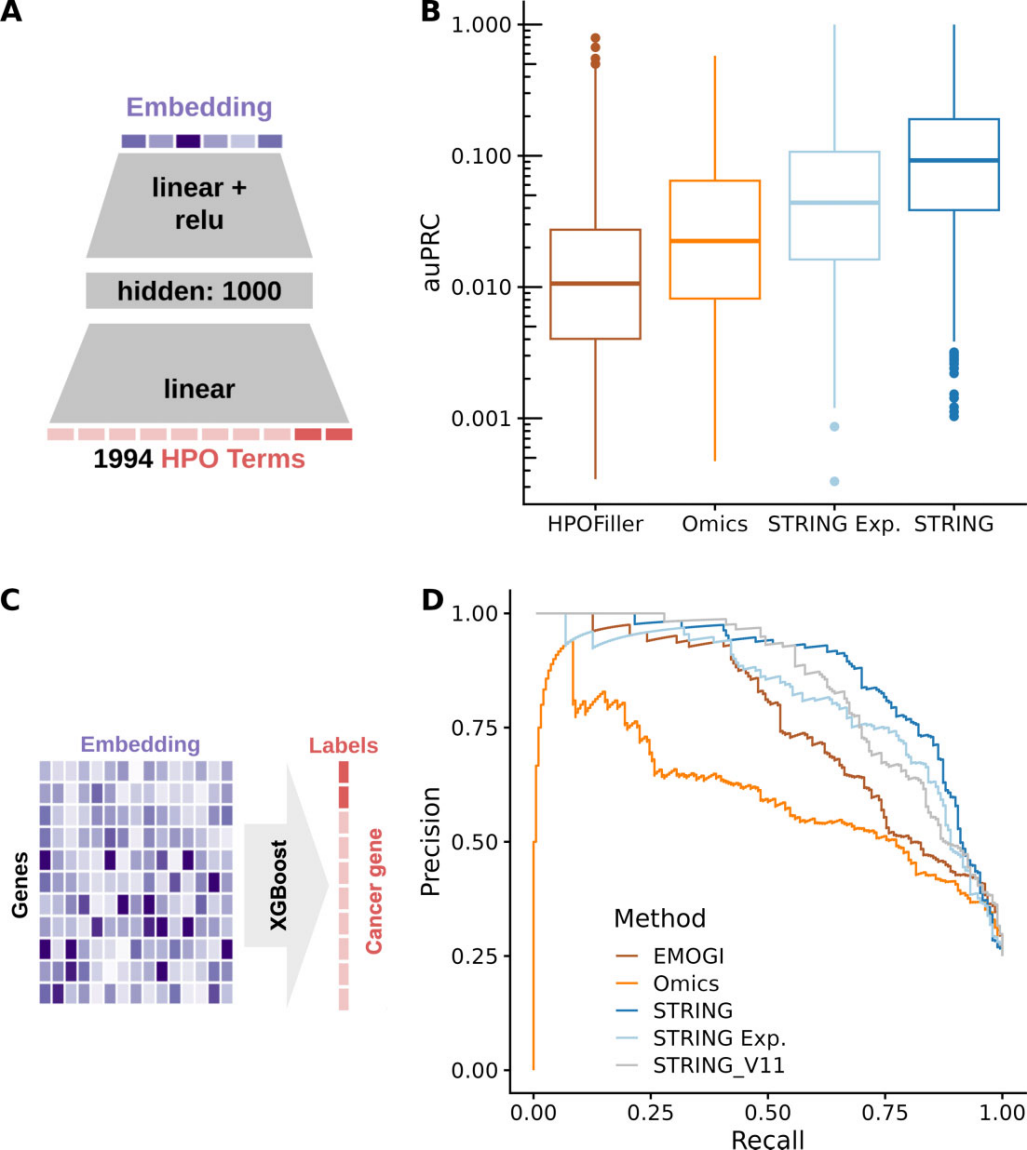
Embeddings improve state-of-the-art models for HPO and cancer gene predictions. (**A**) We predicted phenotype–gene associations annotated in the HPO using a two-layer neural network trained on the embeddings. (**B**) Distribution (boxplot) of performances (auPRC) for each HPO term across four HPO prediction algorithms. All comparisons are significant *P* < 2.19 × 10^−44^ (paired two-sided Wilcoxon test). Centre line = median; box limits = first and third quartiles; whiskers span all data within 1.5 interquartile ranges of the lower and upper quartiles. (**C**) We predicted cancer genes using an XGBoost model trained on our embeddings. (**D**) Precision plotted against recall for the cancer genes.

One advantage of precomputed functional gene embeddings is that baseline predictors can be created with a few lines of code by training simple general-purpose prediction algorithms such as gradient-boosted trees or shallow neural networks taking the embedding as input. We next investigated how such baseline models performed in comparison to dedicated algorithms. To this end, we considered two tasks that have attracted attention in the literature: HPO annotation prediction (4,15,43,53) and cancer gene prediction (35,54). The HPO is a controlled vocabulary of phenotypic abnormalities encountered in human disease. Predicting HPO annotations can aid the genetic diagnosis of individuals suspected of being affected by a rare disorder without a genetic diagnosis in a known disease gene. The state-of-the-art predictor for HPO annotation predictor is HPOFiller, a predictor based on a graph convolutional neural network that uses the STRING protein–protein interaction network as input. Here, we selected HPO terms that are associated with at least 20 genes, resulting in 1994 HPO terms. Next, we trained a simple two-layer multiclass neural network predicting gene annotation for any of the selected HPO terms (Figure 3A). We could not use XGBoost because HPO annotation prediction is a multi-task problem. Nevertheless, a two-layer neural network is a fairly simple model architecture. Thirty per cent of the genes were kept held out for model evaluation. Due to the large number of prediction tasks (1994 HPO terms), using the highly dimensional PoPS embeddings was computationally too intensive and not performed. Nonetheless, we found that predictions using any of our remaining precomputed embeddings outperformed HPOFiller (Figure 3B). As expected, the absolute performance of all models was modest due to the large class imbalance of the HPO prediction tasks. However, we found that all predictions were significantly above background which is approximately at an auPRC of 0.001. The embedding of STRING performed best, followed by the embedding of only the experimental channels of STRING and the Omics embedding. Moreover, our median Area under the ROC-curve (AUC) was consistent with the AUC reported by Deelen *et al.* (4) who used an expression-based embedding (Deelen *et al.*: median AUC = 0.74, Omics: median AUC = 0.75).

Next, we considered the task of predicting cancer genes. We compared our embeddings against the state-of-the-art model EMOGI, a graph convolutional network based on STRING that furthermore integrates omics data from the TCGA dataset as gene features (35). We used the same gene annotations as in EMOGI comprising 783 positive and 2415 negative genes and the same train and test splits (obtained through personal communications). Surprisingly, our gradient-boosted tree trained on the 256-dimension STRING embedding outperformed EMOGI by a significant margin, even when using the same STRING version as EMOGI (auPRC = 0.87 and 0.83 versus auPRC = 0.76, *P* < 2.2 × 10^−16^, paired two-sided Wilcoxon test, Figure 3C, D).

Altogether, these results demonstrate that highly performant baselines can be readily obtained with straightforward applications of standard machine learning algorithms on precomputed functional gene embeddings.

### Benchmarking using association studies

At this point, all benchmarks that we had conducted were based on predicting human-curated gene lists or annotations. Therefore the performance of embeddings containing evidence from the literature, such as STRING or PoPS, might have been overestimated. To assess the utility of the different embeddings for predictions that are not based on human curation, we considered two prediction tasks that are based on mere genotype-phenotype statistical association signals.

**Figure 4.**
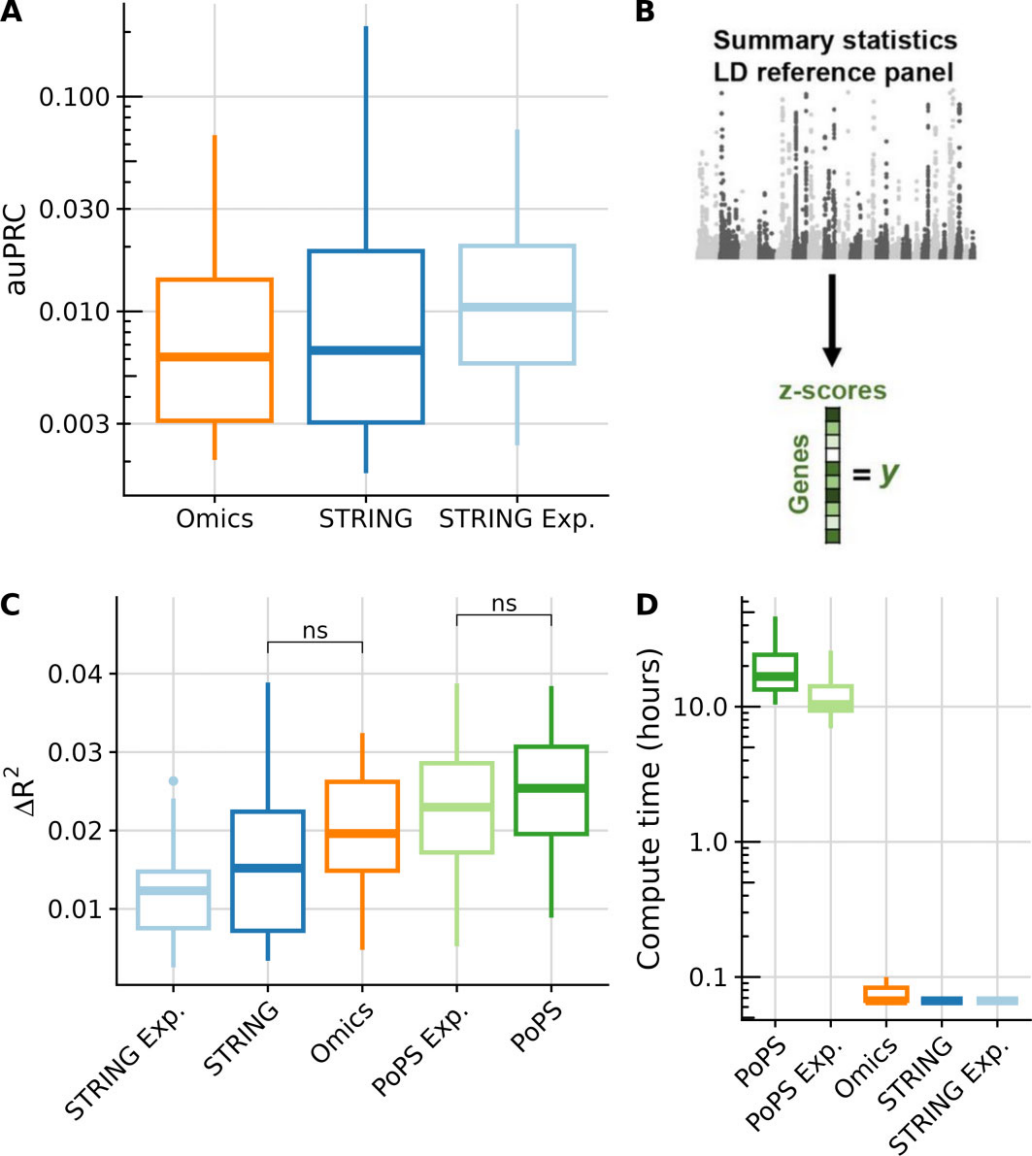
Benchmarking using association studies. (**A**) Distribution (boxplot) across 34 unrelated traits of the area under the precision-recall curve for predicting associated genes in a rare variant association study. None of the embeddings performed significantly better than the others. (**B**) GWAS summary statistics are summarised into z-scores per gene. Adapted from Weeks *et al.* (34) (the rights in the material are owned by a third party). (**C**) Distribution (boxplot) of fit improvements (R2 increase against a model based on covariates only, Materials and methods) for predicting 22 gene-level blood biomarker traits. (**D**) Distribution (boxplot) of computation times for the models shown in (C). For all boxplots, centre line = median; box limits = first and third quartiles; whiskers span all data within 1.5 interquartile ranges of the lower and upper quartiles; paired two-sided Wilcoxon tests were performed in (A), (C) and (D); the significance threshold is *P* < 0.05. In (C) and (D) all pairwise comparisons were significant unless explicitly marked as not significant (ns).

The first task was to predict trait-gene associations reported by Genebass, a rare variant association study performed on the UK Biobank (36,45). To this end, we fitted logistic regressions with elastic regularisation on the embeddings to predict which genes are significantly associated with a given trait. The performance was evaluated using 5-fold cross-validation. As for the HPO prediction, the number of positive genes to train from is very small (Table 1) and therefore the auPRCs are generally low. Nonetheless, all predictors performed significantly better than a random predictor, but none was significantly better than the others (paired Wilcoxon test, Figure 4A). The STRING experimental embedding had a more consistent performance across traits than the STRING embedding (Supplementary Figure S3). The STRING embedding was predictive of some traits such as high-density lipoprotein (HDL) cholesterol and total bilirubin, but performed poorly on most other traits, perhaps because some traits have been more extensively studied than others. Consistent with this interpretation, the Genebass genes associated with the highly studied trait HDL cholesterol were generally well connected by curated and literature links in the STRING network, while they were almost not connected when using the experimental network only (Supplementary Figure S4).

The second task of the kind we considered was to predict gene-level association scores for 22 uncorrelated blood biomarker GWAS studies from the Pan-UK Biobank study (44). Unlike Genebass, this is not a rare variant association study. The gene-level scores in this case capture the overall association of a gene with a trait using common variants. To evaluate the performance of the embeddings, we predicted gene-level scores on held-out chromosomes, similar to the method employed in the PoPS study (34). Predicting gene-level association scores from gene features is a hard task with only a few percent of the overall variance being explained for all traits and models we investigated. Nonetheless, the two PoPS embeddings stood out (Figure 4C). However, the time needed to fit those models was two orders of magnitude longer than the other models (about one day for the PoPS embeddings versus less than five minutes for the Omics and the STRING embeddings on a 16-core server, Figure 4D).

In striking contrast to the human-curated gene list tasks considered above, the STRING embedding was not performing best on predicting gene-level association scores, indicating that annotation inequality and possibly information leakage drives the STRING embedding performance on predicting curated gene lists. Nevertheless, the STRING embedding still did not perform significantly worse than the Omics embedding. We investigated instances manually. A GWAS performed in 2008 identified six loci associated with triglycerides (55). That study is listed as evidence in the STRING literature channel for the edge between the genes *TBL2* and *GALNT2*, two of the six loci linked to triglycerides. Hence, our STRING embedding contains edges based on an older GWAS, from which we are predicting a more recent GWAS on the same trait. This observation showcases the difficulty of establishing benchmarks devoid of information leakage when using literature-derived input features in genomics.

### Effect of annotation inequality

**Figure 5.**
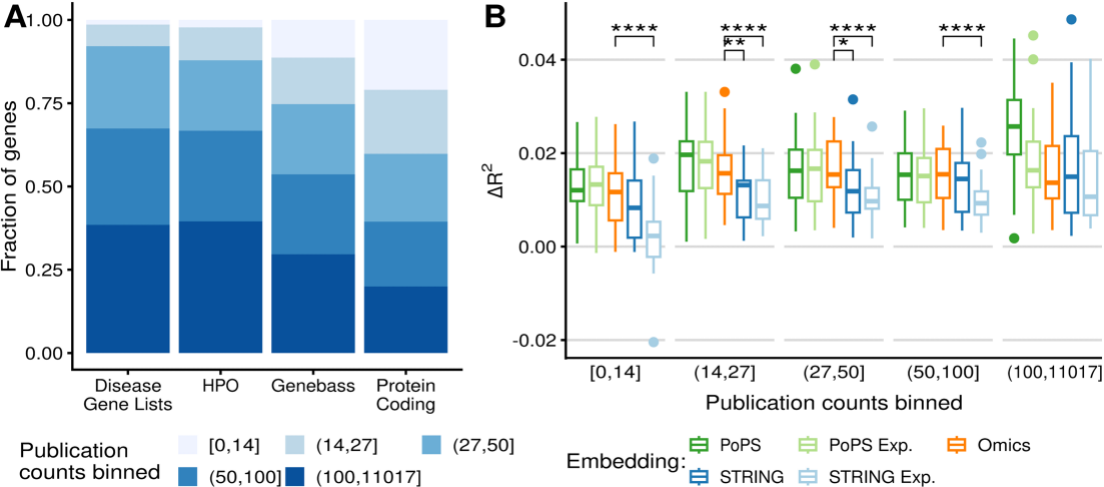
Benchmarks underlie publication biases. (**A**) Fraction of genes in five equally-sized publication bins for each gene prediction task. (**B**) Distribution (boxplot) of fit improvements (*R*^2^ increase against a model based on covariates only, Materials and methods) for predicting 22 gene-level blood biomarker traits stratified by the number of publications per gene. For all boxplots: Centre line = median; box limits = first and third quartiles; whiskers span all data within 1.5 interquartile ranges of the lower and upper quartiles; points are plotted for any points outside that range. Paired two-sided Wilcoxon tests were performed between the Omics and the two STRING embeddings. Only significant *P*-values are shown (*P < 0.05, **P < 0.01, ***P < 0.01, ****P < 1 × 10^−4^).

The STRING embeddings performed worse than the Omics embedding at gene-level GWAS signal prediction (Figure 4C) perhaps because they suffer more from annotation inequality. To assess the effect of annotation inequality, we next investigated the number of publications per gene in each benchmark (Methods). The curated disease-gene lists and HPO terms appear in more publications than all protein-coding genes (Figure 5A). Almost no gene in those lists had fewer than 15 publications. The Genebass genes were comparatively less biassed for high publication numbers. Nonetheless, Genebass genes appeared in more publications than random protein-coding genes. Perhaps, as genes associated in rare variant association studies tend to be constrained (56) and thus may generally play important biological roles, they could have already been the subject of multiple studies.

For the prediction of GWAS signals, we stratified the predictions into five equally-sized groups of genes based on their number of publications. In contrast to Omics and PoPS experimental embeddings, the performance of both STRING embeddings depended on the number of publications, with poorer fits for the genes named in fewer publications (Figure 5B, Spearman Rank Correlation test between publication bin index and Δ*R*^2^, Omics: *P* = 0.22, PoPS experimental: *P* = 0.14, STRING: *P* = 8 × 10^−4^, STRING experimental: *P* = 2 × 10^−6^). Generally both STRING and STRING experimental performed worse than the Omics embedding for genes named in less than 50 publications (Figure 5B). Notably, the STRING experimental embedding had almost no predictive power on the genes with fewer than 15 publications, perhaps because the evidence of the STRING experimental embedding is often based on single-gene studies. For the PoPS embedding, we observed a dependence (Spearman Rank Correlation test between publication bin index and Δ*R*^2^, *P* = 4 × 10^−4^), as the performance for the most studied genes was higher than for all other publication count bins. Part of this higher performance could be driven by data leakage, namely that the major genes for those traits may have been already reported to be functionally related in pathway databases based on earlier GWAS studies, as we pointed out above for the example of STRING. Another contribution to this higher performance could be that, generally, the most studied genes are biologically more important and thus are more likely to be involved in any given trait. The PoPS embedding, which combines annotation database and large-scale expression data allows performing well for all publication count bins. Taken together, these analyses underscore the relevance of using functional annotations based on genome-wide assays.

### Impact of embedding dimension and data modality on prediction performance

We set out to examine the potential impact of embedding dimensions on prediction performance. To do so, we systematically generated STRING embeddings as described in Methods but changed the dimensions from 256 to 48, 128 and 512. We additionally computed PCA projections of each of the PoPS embeddings with 128, 256 and 512 dimensions. We then repeated the gene list, genebass and MAGMA prediction tasks using all these alternate embedding dimensions. Through the analysis, we discerned that the disparities in prediction performance stemming from the embedding dimension were relatively modest when compared to the more prominent variances attributed to the data modality (Supplementary Figures S5–S7). Moreover, we observed that 256 dimensions frequently gave the best result across embeddings and tasks and thus used 256-dimensional embeddings for Omics, STRING and STRING experimental for our main figures.

We also evaluated the contribution of each data modality in the Omics embedding through ablations. In these ablations we removed each data modality individually while generating the embedding, resulting in three new embeddings. We compared these embeddings to the full Omics embedding on the same three tasks as before. We found the GTEx data modality to consistently exert the most influence across tasks (Supplementary Figure S8).

## Discussion

In this study, we have created multiple functional gene embeddings from different functional genomics data modalities. These embeddings can be readily used as precomputed numerical representations of gene function in machine-learning models. They can also be manually investigated to generate functional hypotheses for unannotated genes. Using these functional gene embeddings, we demonstrated that one can easily build predictors with off-the-shelf machine learning algorithms and achieve state-of-the-art predictive performance. Furthermore, leveraging gene-trait associations found in GWAS, we have shown that embeddings based on genome-scale experimental data are less prone to annotation inequality biases than embeddings based on curated databases, text mining, or experimental data from single-gene studies. All our embeddings, benchmarks and code are made available as free resources.

Our study has limitations. First, we had to use different embedding algorithms to process the original PoPs embedding, which is a complete matrix, our omics dataset, which is a set of matrices over slightly different gene sets, and STRING, which is a network. Perhaps, some of the differences we reported shall be attributed to the algorithms and not to the data modalities per se. Moreover, the conclusions drawn from curated gene list benchmarks differed from those of the genetic association benchmarks. It remains to be determined whether these discrepancies are due to the suitability of certain embeddings for particular tasks or to limitations in our benchmarks. Disease-gene lists and ontologies produced by manual curation are inherently biased by the curation process in contrast to benchmarks based on GWAS signal. However, we showed that there remains a small risk of data leakage due to the results of earlier GWAS being integrated into literature-based gene networks. While we have demonstrated that these circularities exist in the STRING literature channel, we suspect that the problem is much more severe, as to some extent the same studies that enter the literature channel are integrated by curators into pathway databases and manually curated interaction networks. The presumably least literature-biassed task of all our benchmarks is the prediction of trait-gene associations derived from Genebass, as the Genebass study is the first large-scale study of its kind, and no other rare variant association study has reported a similar number of associations that could have entered the current STRING version. However, these data may be included in the network on the next STRING version and will no longer serve as an unbiased benchmark. As new data releases will introduce circularity into the benchmarks, it will be important to develop new strategies for unbiased evaluation of gene embeddings. This could include predicting other systematic measurements from new functional screens at the level of single genes or pairs of genes such as measured protein interactions or epistastic interactions.

Given these limitations, we cannot provide a definitive recommendation on which embeddings are best suited for practical applications. Acknowledging this, we expect the STRING embedding to perform best for tasks related to well-studied traits and genes, where exploiting gene-gene interactions and neighbourhoods is relevant. However, we recommend avoiding having literature evidence entering both the input and the output. We expect the Omics embeddings to perform better than STRING for tasks on less studied traits and genes. In conclusion, we recommend evaluating all developed embeddings before applying them to specific tasks. As we have shown, evaluations of the respective potential of the different embeddings can be done rapidly with little computational and engineering effort.

Our embeddings enabled state-of-the-art predictive performance to be achieved on all of our benchmarks. We have therefore not extensively optimised them. Additional improvements are probably achievable, for example by further hyperparameter optimisation, by including additional data modalities, or by jointly embedding gene networks and gene features using techniques such as graph convolutional networks (57). Further, it will probably be helpful to update the embeddings regularly by including novel data. To this end, we hope that the benchmarking strategies that we developed here will allow the community to further improve the embeddings.

Representation learning is an appealing approach for reducing the complexity and abstracting the specificity of diverse data modalities allowing easy use of the combined information derived from those datasets across research areas. The functional gene embeddings generated in this study have the potential to lower the barrier to entry for biomedical modellers lacking genomics expertise. For instance, by leveraging our embeddings, researchers could more easily build models linking genetic alterations to electronic health records or imaging, or embeddings thereof (58,59), facilitating the discovery of novel connections across different types of biomedical data.

## Supplementary Material

lqad095_Supplemental_Files

## Data Availability

No new data was generated for this study. For a description of the used datasets, refer to Materials and Methods. All scripts required to reproduce the figures in this manuscript are available at Zenodo, https://doi.org/10.5281/zenodo.8375636.
