# Supplementary material for: Evaluation of input data modality choices on functional gene embeddings
Source: NAR Genom Bioinform. 2023 Nov 2;5(4):lqad095. doi: 10.1093/nargab/lqad095 (PMC10629286; doi:10.1093/nargab/lqad095)
Supplement: lqad095_Supplemental_Files [file lqad095_supplemental_files.zip › Revision_of_Supplementary_Figures.pdf]

# Evaluation of input data modality choices on functional gene embeddings - Supplementary Figures

Felix Brechtmann[1,2], Thibault Bechtler[1], Shubhankar Londhe[1], Christian Mertes[1,3,4], Julien Gagneur[1,4,5,*]

1. TUM School of Computation, Information and Technology, Technical University of Munich, Garching, Germany
2. Munich Center for Machine Learning, Munich, Germany
3. Munich Data Science Institute, Technical University of Munich, Garching, Germany
4. Institute of Human Genetics, Klinikum rechts der Isar, School of Medicine, Technical University of Munich, Munich, Germany
5. Institute of Computational Biology, Computational Health Center, Helmholtz Zentrum München, Neuherberg, Germany

*Corresponding author. Email: gagneur@in.tum.de

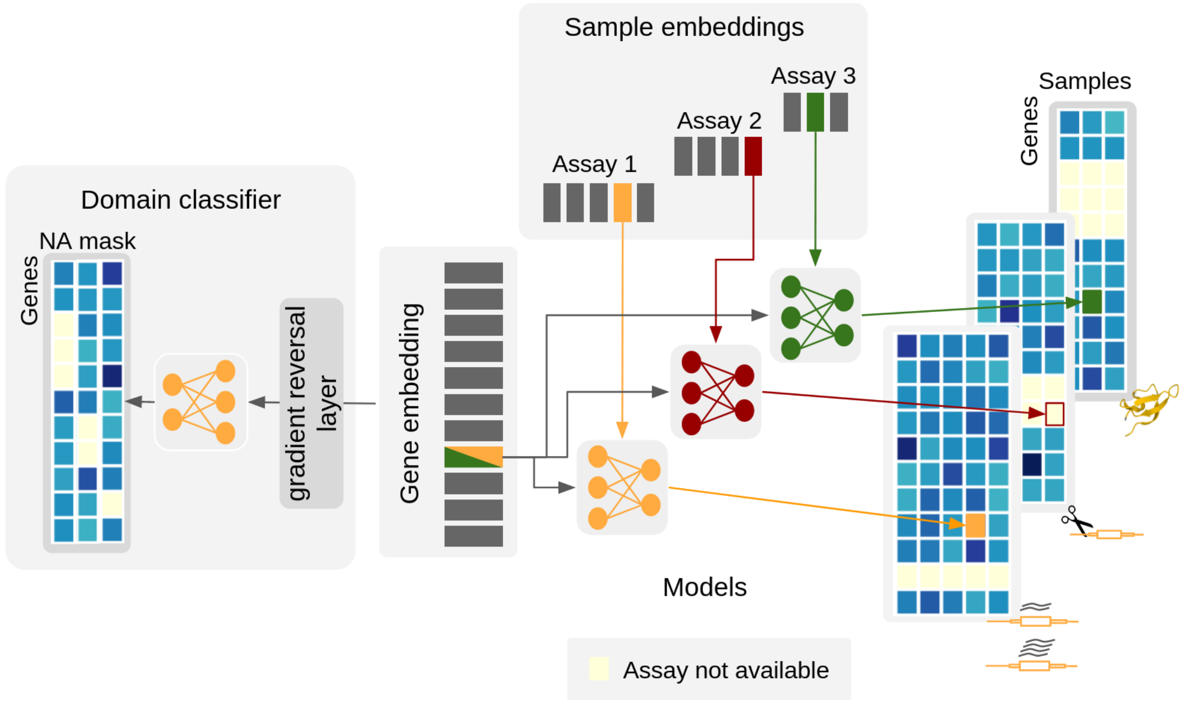


**Figure S1: Architecture of the variational tensor factorisation model used to create the Omics embedding.** The Omics embedding is created by condensing information from genome wide assays into embeddings representative of gene function. One assay agnostic gene embedding (center) and multiple assay specific sample embeddings (top) are optimised to reconstruct the observed data per assay (right). Additionally, a domain classifier with a gradient reversal layer ensures that the embedding does not learn the missingness of the dataset. The latent embeddings are learned by backpropagation through multiple models. Genes absent from assays are not considered during backpropagation.

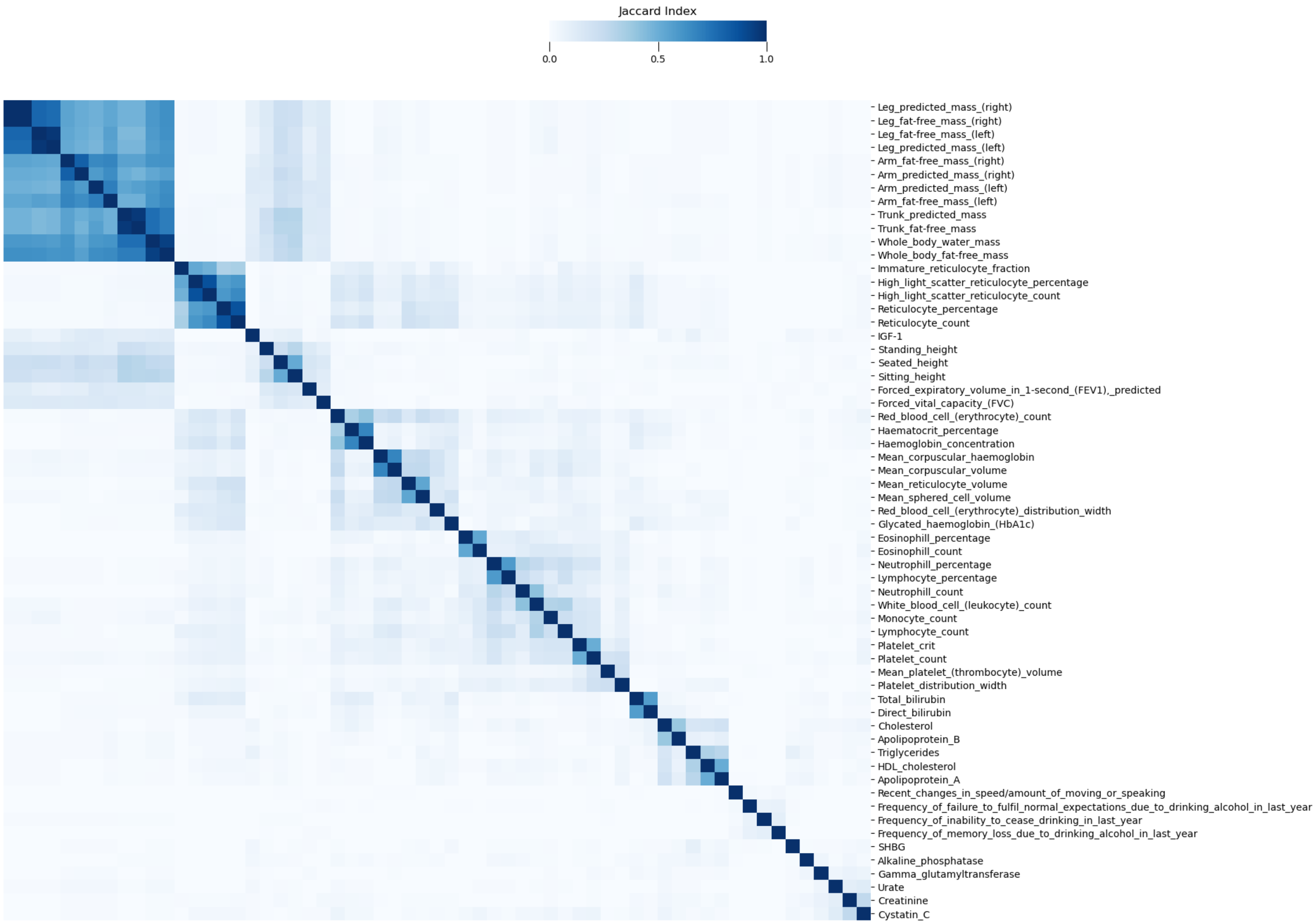


**Figure S2: Overlap of rare variant gene-trait associations for 61 UK Biobank traits.** Pairwise Jaccard indices between all 61 traits. The Jaccard index was calculated as the fraction of the number of genes associated with both traits over the number of genes associated with either trait. Hierarchical clustering was performed using the fcluster package in scipy with a threshold of 0.7 resulting in 34 clusters. One trait was chosen from each cluster for further analysis.

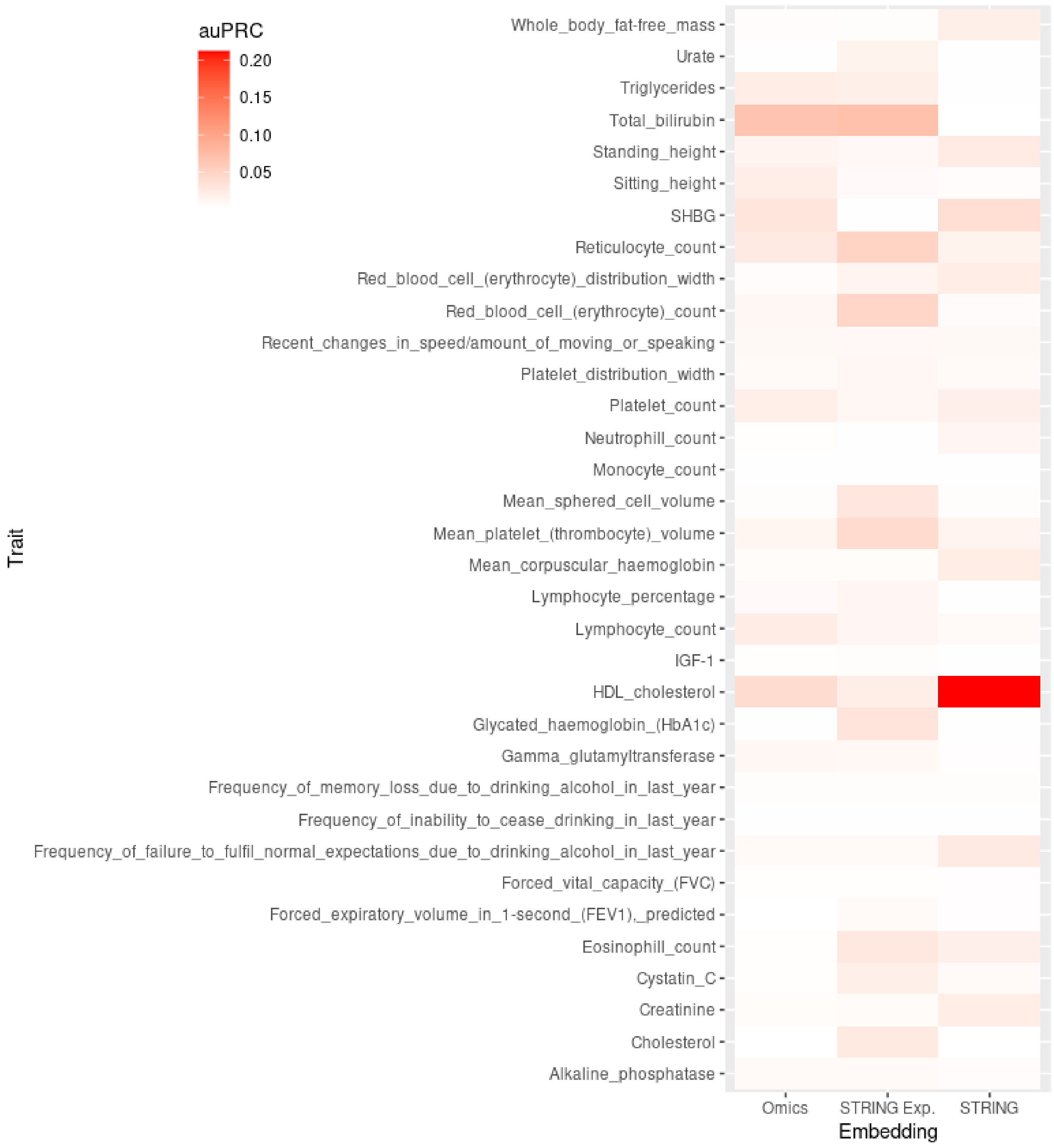


**Figure S3: Heatmap of auPRC scores on 34 traits chosen from a rare variant association study on the UK Biobank.** Distribution of auPRC scores across 34 selected traits for predicting associated genes in a rare variant association study. The predictions were made using logistic regression models with elastic regularisation on the embeddings to predict which genes are associated with a given trait. The STRING experimental embedding is more consistent across traits, whereas the STRING embedding performs well on highly studied traits such as HDL (high-density lipoprotein) cholesterol and total bilirubin.

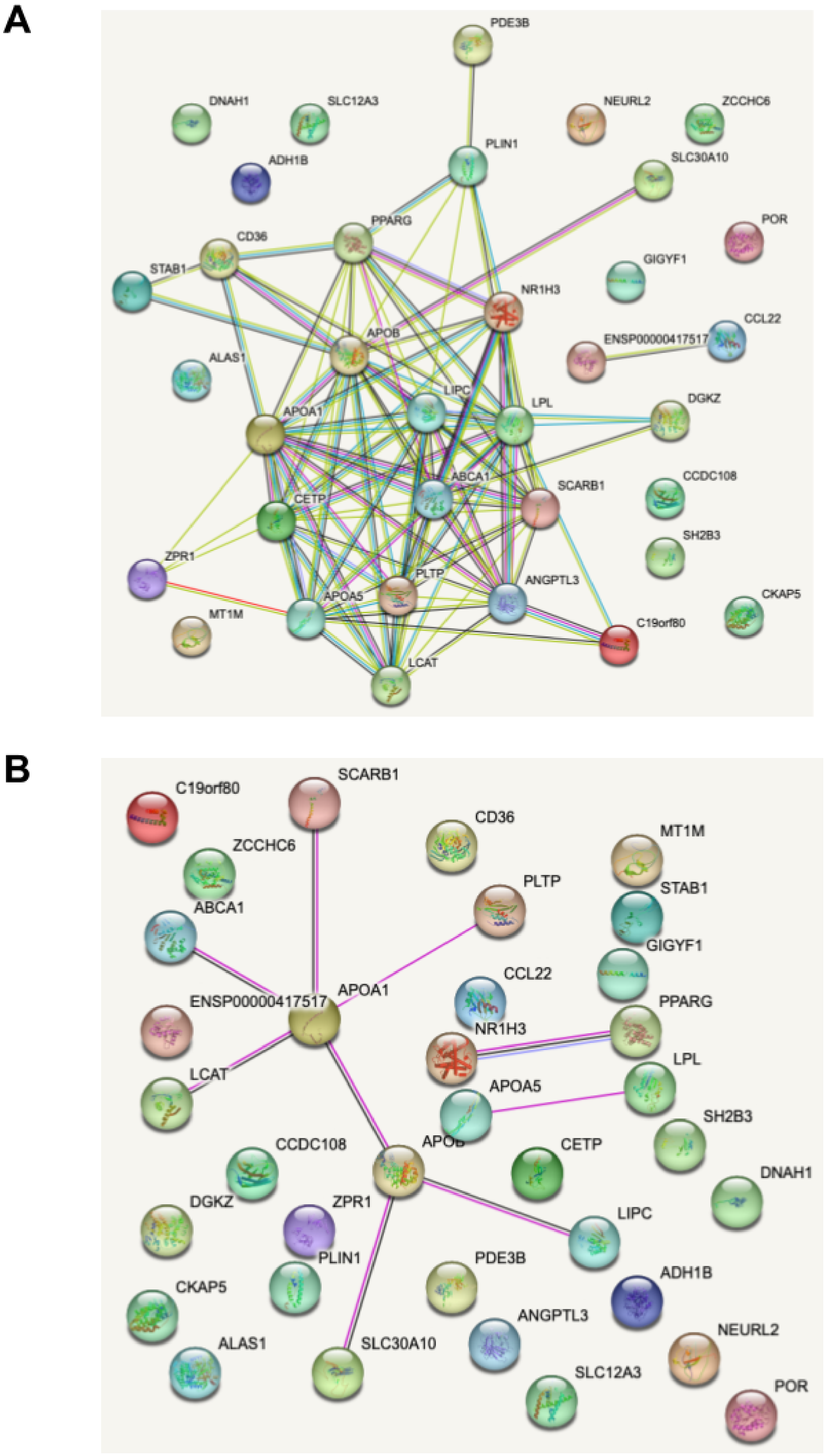


**Figure S4: Difference in graph connectivity of genes associated with HDL in STRING is indicative of bias towards well-studied traits. (A)** STRING network of the 36 genes associated with HDL cholesterol in Genebass. These genes show high connectivity in STRING when using human-curated and literature sources to create edges. **(B)** STRING network of the genes associated with HDL cholesterol without human-curated and literature sources. The connectivity between genes is noticeably reduced when using STRING with experimental sources only.

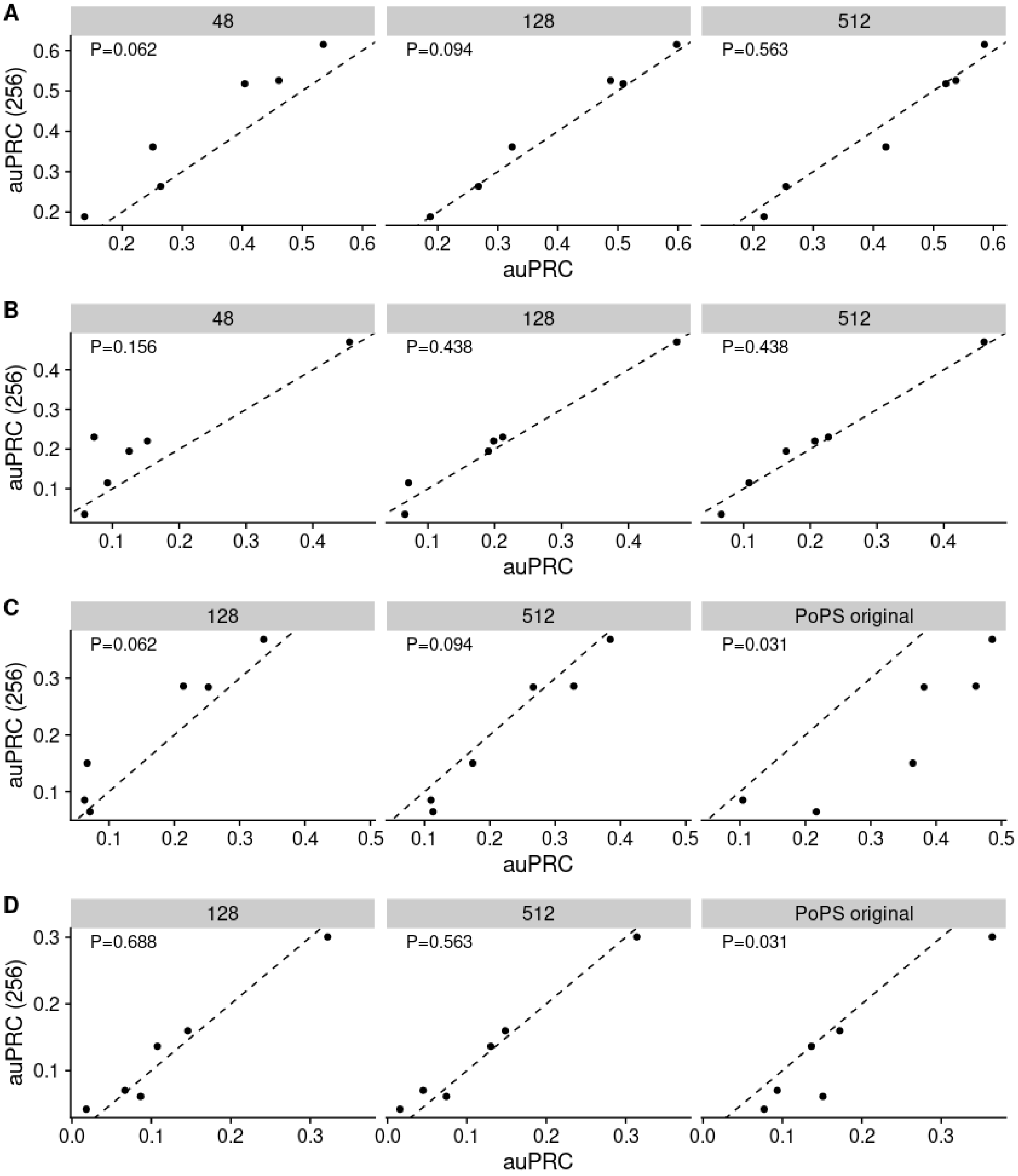


**Figure S5: Impact of embedding dimension on disease-gene prediction. (A-D)** Predictive performance (area under the precision-recall curve, auPRC) for the 256 dimensional embeddings (y-axis) plotted against performances (x-axis) using alternative embedding dimensions (rows) for each embedding (A) STRING, (B) STRING Exp, (C) PoPS and (D) PoPS Exp. Overall no large deviations can be observed (Wilcoxon paired test). The clearly better performance for PoPS in panel © is likely due to data leakage.

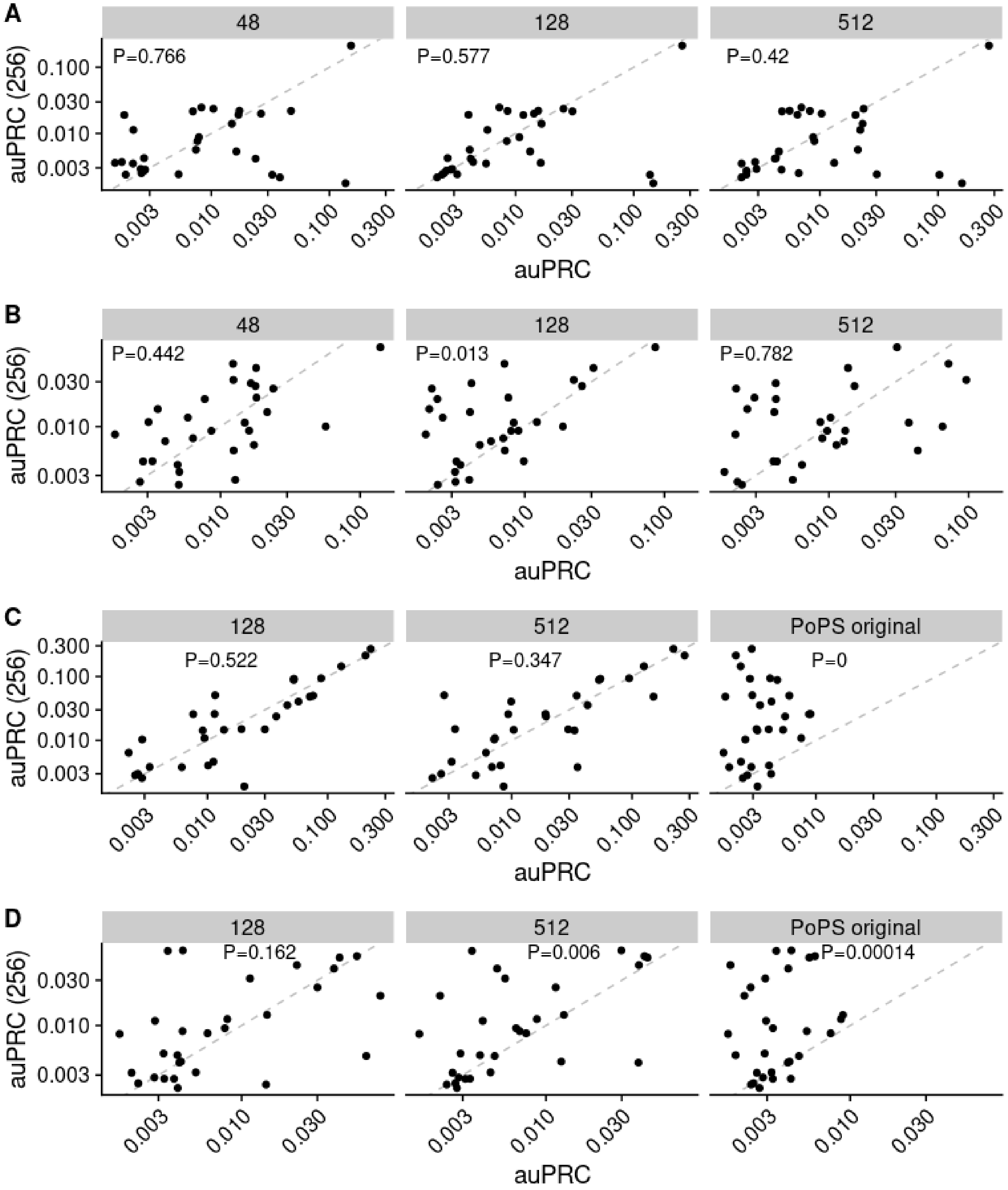


**Figure S6: Impact of dimension on Genebass gene-trait association prediction. (A-D)** The predictive performance (area under the Precision-Recall curve, auPRC) for the 256 dimensional embedding (y-axis) compared against embedding performances (x-axis) using alternative dimensions (rows) on 29 traits for each embedding (A) STRING, (B) STRING Exp, (C) PoPS and (D) PoPS Exp. The auPRC is computed on the predictions of an XGBoost predictor on the 48, 128, 256, and 512 dimensional embeddings. A logistic regression with ElasticNet regularisation was used on the original PoPS expression matrix as XGBoost was infeasible to compute. The fit performance is independent of the dimensionality in almost all cases (Wilcoxon paired test).

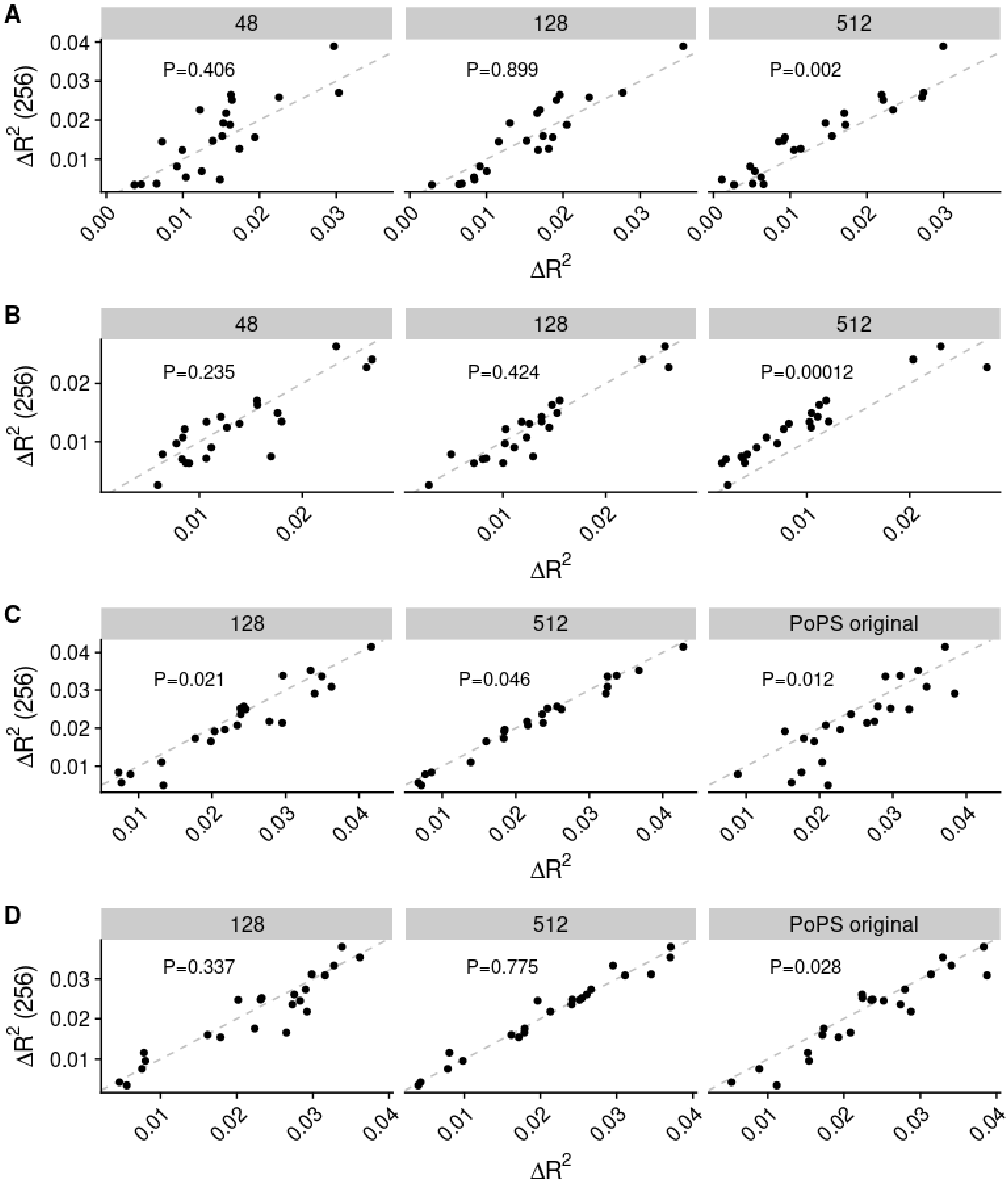


**Figure S7: Benchmarking using association studies dimensionality analysis. (A-D)** Fit improvements (y-axis) based on 256-dimensional embeddings ($R^2$ increase against a model based on covariates only, Methods) for predicting MAGMA z-scores of 22 gene-level blood biomarker traits are plotted against the same improvements (x-axis) using alternative dimensions (rows) for each embedding (A) STRING, (B) STRING Exp, (C) PoPS and (D) PoPS Exp. The fit performance is independent of the dimensionality in almost all cases (Wilcoxon paired test).

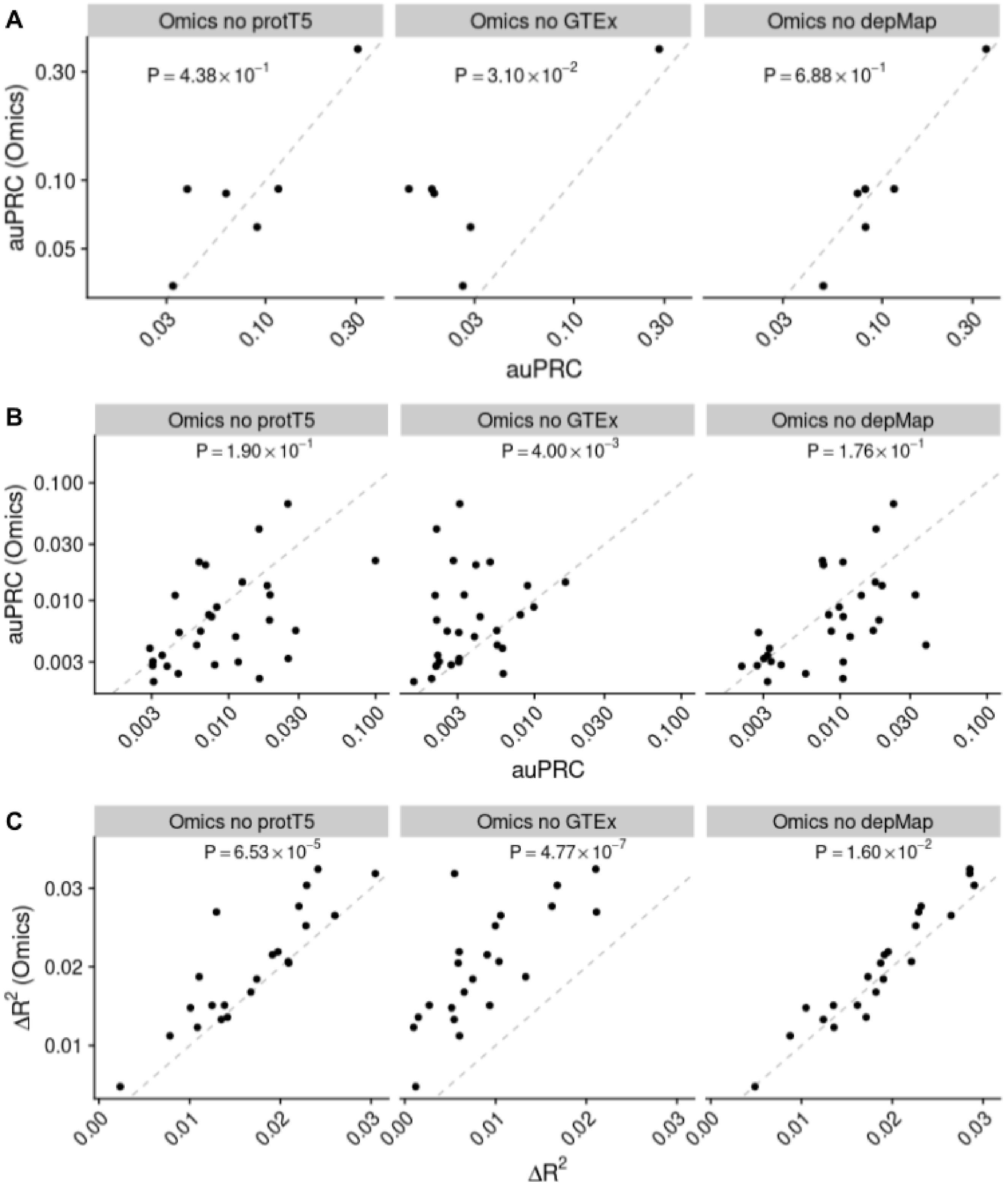


**Figure S8: Contribution of data modalities in the Omics embedding through ablation analysis. (A)** Comparison of disease-gene prediction performance of the Omics embedding compared to variants of the Omics embedding created by omitting a data modality during fitting of the embedding. Only the removal of GTEx has a significant effect on the prediction performance (Wilcoxon paired test *P*-value < 0.05). **(B)** Same as in (A), but on Genebass trait-gene association prediction. Here, only the removal of GTEx has a significant effect on the prediction performance (Wilcoxon paired test *P*-value < 0.05). **(C)** Same as in (A), but on MAGMA score prediction. Removing any of the data modalities yields a significantly worse performance, however, the effect is most pronounced on the removal of GTEx.
